# Dexmedetomidine for improving postpartum sleep quality following cesarean section in women: a randomized controlled trial

**DOI:** 10.3389/fmed.2026.1823100

**Published:** 2026-04-30

**Authors:** Jie Gao, Chen-Chen Hu, Xin-Yue Wang, Meng-Meng Li, Yong-Lu Pan, Rui Yang

**Affiliations:** 1 Department of Anesthesiology, The First Affiliated Hospital of Anhui Medical University, Hefei, China; 2 Department of Anesthesiology, Union Hospital, Tongji Medical College, Huazhong University of Science and Technology, Wuhan, China

**Keywords:** cesarean section, dexmedetomidine, pain score, parturients, sleep quality

## Abstract

**Background:**

Perinatal sleep disturbance is highly prevalent among women undergoing elective cesarean section. Dexmedetomidine has been proven effective in improving sleep quality in patients undergoing general surgery, and this study aimed to investigate its potential efficacy in improving postoperative sleep quality in the elective cesarean section population.

**Methods:**

A total of 68 parturients scheduled for elective cesarean section were randomly allocated to the dexmedetomidine group (Group D) and the control group (Group C). Following fetal delivery, Group D received an intravenous bolus infusion of 0.5 μg/kg dexmedetomidine within 15 min, while Group C was administered an equal volume of normal saline as a control. Outcome measures included sleep quality scores at 1st, 2nd, 3rd day and 6th week postoperatively, STAI score at 1st, 2nd and 3rd day postoperatively, resting and activity-related pain scores at 24 h postoperatively, 24-h consumption of patient-controlled PCEA, the incidence of intraoperative shivering, EPDS scores at 6th week postoperatively and NBNA scores within 24 h/48 h after surgery. All outcomes were systematically recorded and compared between the two groups.

**Results:**

Group D had significantly higher sleep quality scores than Group C at the first postoperative night and 6th week postoperatively (*p* < 0.001 and *p* = 0.01, respectively). At 24 h postoperatively, Group D exhibited lower activity-related pain scores and reduced 24-h PCEA consumption compared with Group C (both *p* < 0.001), and postoperative anxiety levels were significantly decreased in Group D on the first postoperative day (*p* < 0.05). No significant difference was observed in EPDS scores between the two groups at 6th week postoperatively. Additionally, the incidence of intraoperative shivering was markedly lower in Group D than in Group C (*p* < 0.001) and the NBNA scores were similar between Group D and Group C.

**Conclusion:**

For parturients undergoing elective cesarean section, intraoperative intravenous bolus infusion of dexmedetomidine exerts a positive effect on improving sleep quality at the first postoperative night and 6th week postoperatively, as well as reducing activity-related pain scores at 24 h postoperatively.

**Clinical trial registration:**

Chinese Clinical Trial Registry (ChiCTR2500106212) https://www.chictr.org.cn/showprojEN.html?proj=280761.

## 1 Introduction

Adequate sleep is a cornerstone of optimal postoperative recovery for parturients, as it underpins physical healing, neuroendocrine regulation and psychological status in the postpartum period (1). Yet poor sleep quality is a highly prevalent and well-documented issue among postpartum women, with over 60% reporting significant sleep disturbances in the early postpartum phase (2, 3). For most new mothers, sleep duration and quality reach their nadir in the immediate postpartum weeks, characterized by frequent awakenings, shortened sleep cycles and reduced sleep efficiency, with a gradual return to baseline levels only over the subsequent six months (4). Cesarean section is a commonly selected mode of delivery among parturients, and China has previously reported the highest cesarean section rate worldwide (5, 6). Notably, postoperative sleep disturbances are markedly more common and severe in cesarean section parturients, driven by the synergistic effect of multiple interrelated physical and psychological factors (4, 7, 8).

A growing body of evidence has identified key contributors to impaired postpartum sleep in this specific cohort, including pre-existing poor preoperative sleep quality, persistent postoperative surgical pain, disruptive environmental conditions in the general ward, and the inherent psychological stress and anxiety associated with childbirth and cesarean surgery (8, 9). Suboptimal sleep exerts a profound and multifaceted impact on the physical and mental health of postpartum women (1). Systematic reviews have confirmed an association between daily sleep duration of fewer than 5 h and postpartum weight retention (3, 10), a condition that impedes physical recovery and may increase long-term risks of metabolic dysfunction (3). Furthermore, poor postpartum sleep is widely recognized as a major risk factor for postpartum depression (11), a common and debilitating postpartum complication.

In clinical practice, several non-pharmacological interventions have been validated as effective for improving postpartum sleep, including sleep health education, cognitive behavioral therapy for insomnia, relaxation training, and targeted modifications to the sleep environment (12–14). While pharmacological therapy remains the first-line approach for managing postoperative sleep disturbance in the general population, both postpartum women and clinicians alike prioritize non-pharmacological strategies for this vulnerable group (15). This clinical preference stems from well-founded concerns regarding potential teratogenic effects of medications and their transfer into breast milk, which could pose risks to newborns in the critical early postnatal period (16). Consequently, a highly cautious and evidence-based approach is imperative when investigating the potential efficacy of pharmacotherapies for improving sleep quality in postpartum women.

Dexmedetomidine, a highly selective *α*₂-adrenergic receptor agonist, has demonstrated robust pharmacological efficacy in enhancing perioperative sleep quality among surgical patients (17–19). In our previous studies, we observed that intraoperative dexmedetomidine infusion improved sleep quality and upregulated melatonin secretion in patients undergoing lobectomy during the immediate postoperative night (20, 21). Despite its well-documented benefits for perioperative sleep, however, the clinical application of dexmedetomidine in obstetrics remains remarkably limited, with an extreme paucity of literature investigating its use during the perinatal period (14, 22). A recent study has identified a potential role for epidurally administered dexmedetomidine in ameliorating postoperative sleep disturbance in women undergoing elective cesarean section, which attributed this effect to the agent’s ability to optimize analgesic efficacy and reduce ropivacaine consumption when used as an epidural adjuvant (17). This emerging evidence highlights a key unresolved clinical inquiry that warrants further investigation: the potential sleep-improving effects of intravenous dexmedetomidine infusion administered immediately after fetal umbilical cord clamping, a time point that eliminates the risk of fetal drug exposure, in parturients undergoing cesarean section.

Against this clinical backdrop and research gap, the present randomized controlled study was designed to investigate the effect of a single intraoperative intravenous bolus of dexmedetomidine on sleep quality in parturients undergoing elective cesarean section. We hypothesized that the immediate intravenous infusion of dexmedetomidine after the fetus was exteriorized from the uterine cavity could improve the sleep quality score of the parturients on the first postoperative night.

## 2 Methods

### 2.1 Approval and trial registry

This trial was registered with the Chinese Clinical Trial Registry before enrolling the participants (ChiCTR2500106212). Ethical approval was obtained by the Ethics Committee of The First Affiliated Hospital of Anhui Medical University (PJ-2025-05-20). Informed consent was provided by all patients, and all procedures were performed in accordance with the Declaration of Helsinki.

### 2.2 Participants, treatment and randomization

**Figure 1:**
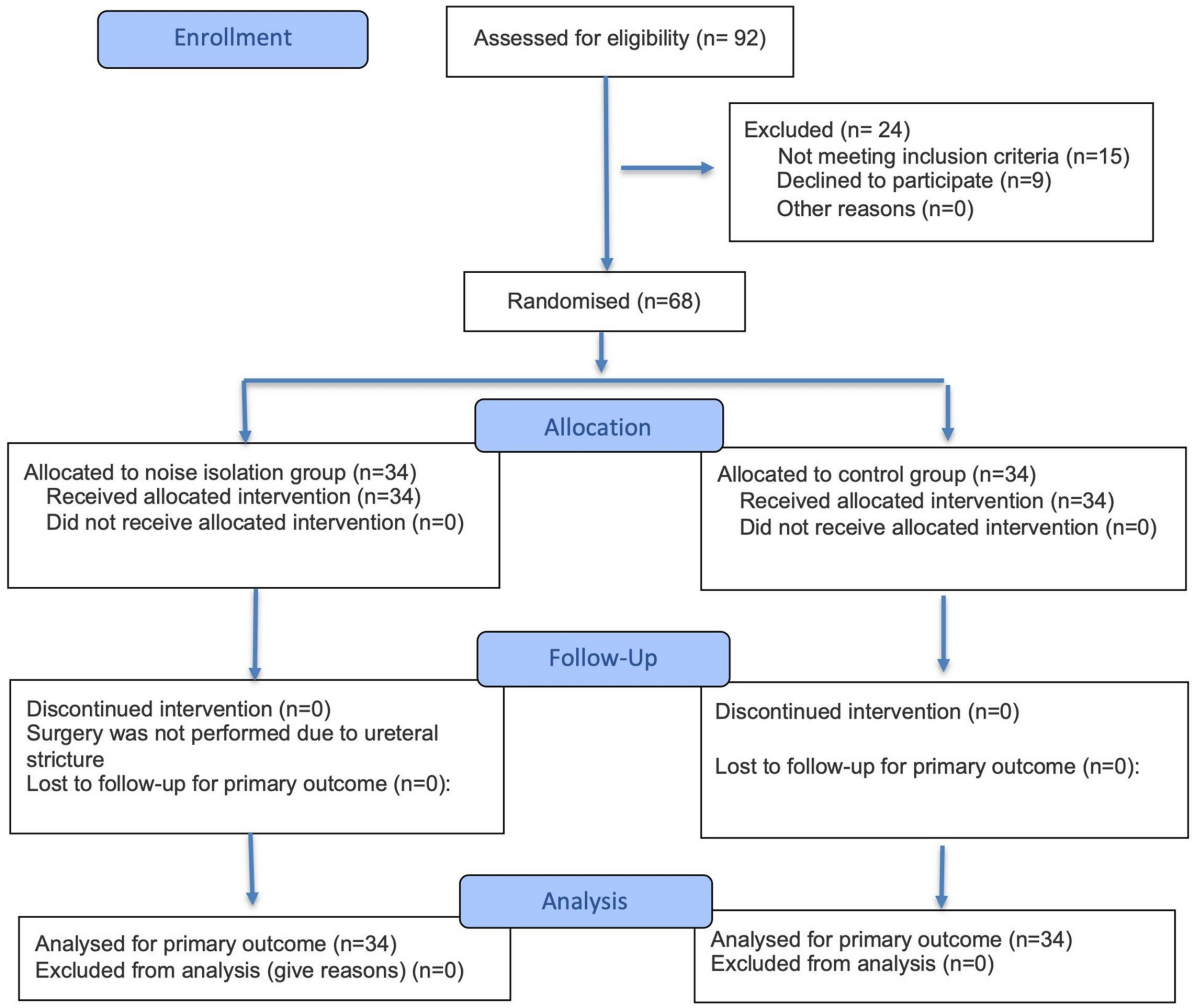
CONSORT 2025 flow diagram. Adapted from: Hopewell et al. (35).

A total of 92 patients aged under 40 years old with American Society of Anesthesiologists (ASA) physical status II–III scheduled for elective cesarean section were screened, and 68 patients completed the study. The Consolidated Standards of Reporting Trials (CONSORT) flow diagram is shown in Figure 1. Exclusion criteria included preoperative drug and alcohol abuse, patients with dementia, contraindications for neuraxial anesthesia, dexmedetomidine contraindications, communication difficulties, and all other factors that might affect the study. All eligible patients were randomly assigned to the dexmedetomidine group (Group D) or the control group (Group C) using a stratified permuted block design. The randomization sequence and block size were computer-generated. Randomization was performed by a research assistant who was independent of the study procedures. All investigators involved in data collection, postpartum follow-up, and statistical analysis, as well as all study participants, remained blinded throughout the trial. Treatment allocation was unblinded only in the event of an emergency threatening maternal or neonatal safety to facilitate appropriate clinical intervention.

All participants received routine monitoring, including heart rate, pulse oxygen saturation, and non-invasive blood pressure after being transferred to the operating room. After the venous access was established, all subjects received epidural anesthesia or spinal anesthesia. The sensory block level was assessed via pinprick testing after anesthesia induction, and surgery commenced once the block level reached the sixth thoracic dermatome. After the fetus was removed from the uterine cavity, patients in Group D received a 0.5 μg/kg dexmedetomidine intravenous infusion within 15 min, while patients in Group C received an equal volume of normal saline infusion. During the infusion of dexmedetomidine, the heart rate fluctuations of all subjects were continuously monitored. Atropine 0.5 mg was administered intravenously for heart rate <50 beats per minute, and dexmedetomidine infusion was discontinued if bradycardia persisted following atropine administration.

Before the end of the surgery, both groups of patients received patient-controlled epidural analgesia (PCEA) to provide postoperative analgesia. The PCEA solution consisted of 200 mg ropivacaine and 50 μg sufentanil in 100 mL saline, with a 2 mL bolus, 30-min lockout, and 2 mL/h background infusion.

Predefined complications were treated according to the study protocol: hypotension (systolic blood pressure below 90 mmHg, reduced by 20% of baseline) was treated by accelerating the infusion rate of sodium lactate Ringer’s solution and intravenous injection of 3–6 mg ephedrine. Bradycardia (heart rate below 50 beats per minute) was treated with intravenous injection of 0.5 mg atropine.

### 2.3 Outcomes measurement

The primary outcome of the study was the sleep quality scores on the first night after surgery (D1, from 22:00 on the day of cesarean section to 08:00 on the first postoperative day), which was evaluated using the Richard Campbell Sleep Questionnaire (RCSQ). The RCSQ is a reliable, patient-reported sleep assessment tool with validated psychometric properties in obstetric patients undergoing cesarean section. It has been widely used in parturients to evaluate the postoperative sleep quality due to its simplicity, high response rate and good correlation with objective sleep monitoring indicators in this specific population (23, 24). The RCSQ consists of six domains. The average score of the first five domains is regarded as the overall sleep quality score. The first five items are as follows: sleep depth, sleep latency, awakenings, sleep efficiency, and sleep quality. The higher the score, the better the sleep quality. The sixth domain is noise perception, where a higher score indicates a quieter environment. Each domain was evaluated using a visual analog scale ranging from 0 to 100 mm.

Secondary outcomes included sleep quality scores on the second night after surgery, the third night after surgery, and the sixth week after delivery, pain scores at rest and during activity 24 h after surgery, and the consumption of PCEA in both groups at 24 h after surgery. Anxiety score was evaluated with the State–Trait Anxiety Inventory (STAI) at baseline (preoperatively) and on postoperative days 1, 2, and 3. At the sixth week postpartum, the researchers screened all the subjects via phone using the Edinburgh Postnatal Depression Scale (EPDS).

In addition, rescue analgesics or antiemetics used during the surgery, perioperative fluid infusion volume and intraoperative blood loss were recorded. Adverse events included the incidence of intraoperative shivering, hypotension, respiratory depression, nausea, and vomiting. Intraoperative shivering was defined as muscle twitching involving the face, chest, and extremities that persisted for at least 15 s. Parturient safety was evaluated by mean heart rate (HR) and mean arterial pressure (MAP) and mean heart rate (HR) during the 15-min dexmedetomidine/saline infusion and Neonatal safety was assessed using at 1- and 5- min Apgar scores and Neonatal Behavioral Neurological Assessment (NBNA).

### 2.4 Sample size calculation and statistical analysis

The sample size calculation for this study was completed using PASS 2021 (NCSS, LLC, Kaysville, UT, USA). The sample size estimation for the formal study was further validated by referencing two published high-quality randomized controlled trials (RCTs) with consistent research endpoints (23, 24). In our pilot study, we included 20 patients who underwent elective cesarean section and were randomly assigned to Group D and Group C. The mean and standard deviation of the sleep quality scores reported by Group D patients on the first night after surgery were 43.8 and 9.2. The mean sleep quality scores reported by Group C patients were 36.0. In this study, 31 patients per group would provide 90% power with a two-sided *α* of 5% to detect differences in the mean sleep quality scores between the two groups. Additionally, we added a power analysis statement (power = 80%, α = 0.05) and clarified that the final sample size was expanded by 10% to account for potential dropouts, the number of patients included in each group increased to 34.

Continuous variables were presented by means and variance or median and quartiles, depending on whether they were normally distributed. The distributions of continuous variables were analyzed by the Kolmogorov–Smirnov normality test. Continuous variables were analyzed using an independent sample t-test or Mann–Whitney U-test. The categorical variable was presented by frequencies and analyzed using Pearson’s chi-square test. Repeated measures analysis of variance was used to compare the differences in postoperative sleep quality scores between the two groups of patients at different time points. A two-sided *p* < 0.05 was deemed statistically significant. All data statistics were completed using SPSS 16.0 version (IBM Corp, Armonk, NY, USA).

## 3 Results

From August 2025 to December 2025, 92 parturients were eligible for screening. Among them, 15 parturients did not meet the inclusion criteria, and 9 parturients refused to participate. A total of 68 parturients were randomly divided into Group D and Group C and included in the final analysis (Figure 1).

**Figure 2:**
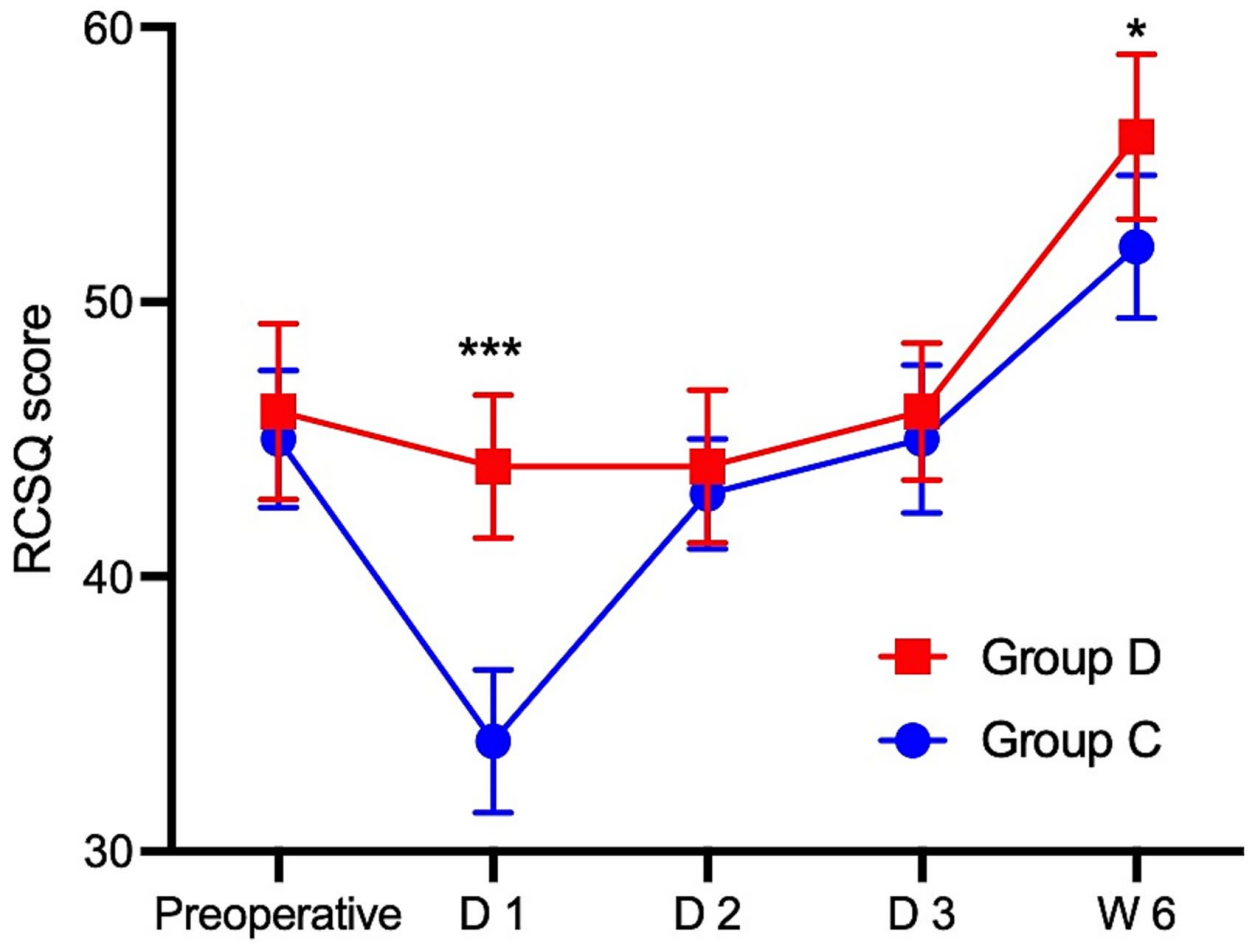
Comparison of RCSQ scores before surgery and within six weeks after surgery between the two groups. *Significant difference (*p* < 0.05) between Group D and Group C; ***Significant difference (*p* < 0.001) between Group D and Group C; n.s no significant difference between Group D and Group C. Data are presented as median (interquartile range [IQR]). D1, first day after surgery; D2, second day after surgery; D3, third day after surgery; W6, six weeks after surgery. RCSQ, Richards-Campbell Sleep Questionnaire; Group D, the dexmedetomidine group; Group C, the control group.

**Figure 3:**
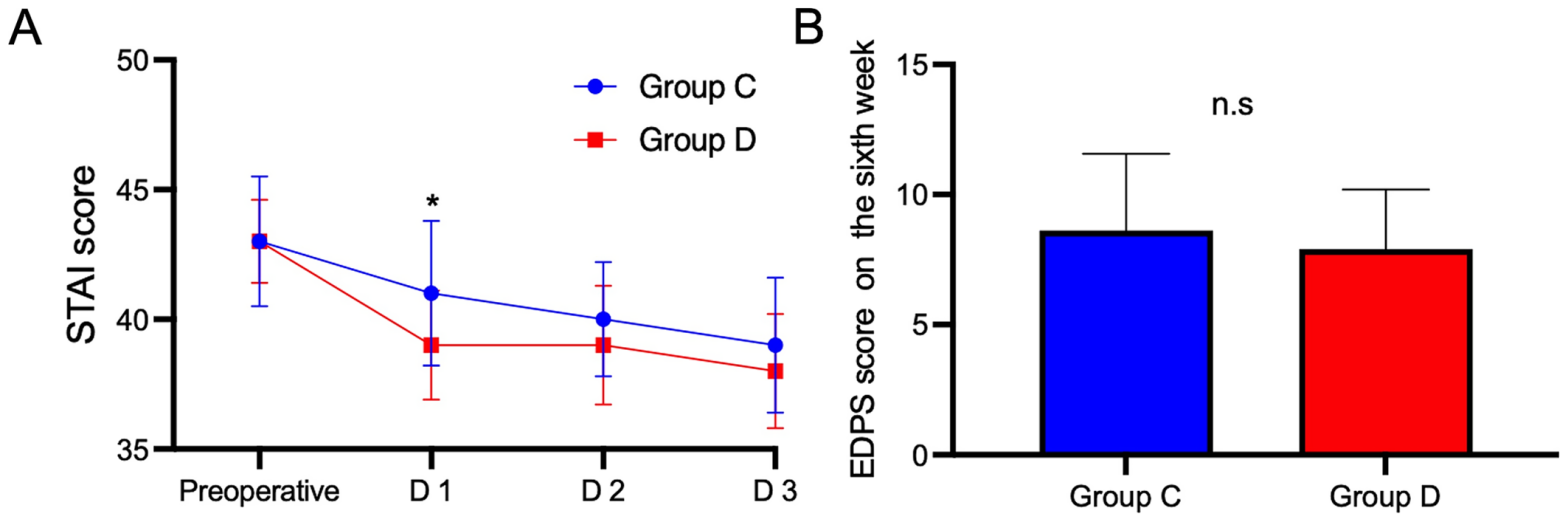
Comparison of STAI scores **(A)** before surgery and within 3 days after surgery and the EPDS scores **(B)** on the sixth week between the two groups. *Significant difference (*p* < 0.05) between Group D and Group C; n.s no significant difference between Group D and Group C. Data are presented as median (interquartile range [IQR]). D1, first day after surgery; D2, second day after surgery; D3, third day after surgery. STAI, State–Trait Anxiety Inventory; EPDS, Edinburgh Postnatal Depression Scale, Group D, the dexmedetomidine group; Group C, the control group.

The demographic data and baseline data of the two groups of patients were comparable (Table 1). As shown in Figure 2, there was no significant difference in baseline sleep quality scores between Group D and the Group C (46 ± 3.2 vs. 45 ± 2.5, *p* > 0.05), the RCSQ scores of Group D patients on the first night after surgery (sleep quality scores of patients in Group D decreased from 46 before surgery to 44 on the first night after surgery, and those of patients in Group C decreased from 44 before surgery to 34, 44 ± 2.6 vs. 34 ± 2.6, *p* < 0.001) and sixth week after surgery were higher than those of Group C (56 ± 3 vs. 52 ± 2.6, *p =* 0.01). The RCSQ scores of the two groups of patients on the second night after surgery and the third night after surgery were comparable with those of the patients in Group C (Figure 2, both *p* > 0.05). To eliminate the interference of time on the results, the overall *p* value for the sleep quality score in each period between the two groups was calculated, and a difference was found between the groups (*p <* 0.0001), indicating that the sleep quality scores of the two groups of patients after surgery increased progressively over time. Twenty-four hours after surgery, the pain scores during activity (Table 2 1 vs. 2, *p* < 0.001) and PCEA consumption of patients in Group D were lower than those of patients in Group C (Table 2, 50 vs. 56, *p* < 0.001). Preoperative STAI scores were similar between Group D and Group C (Figure 3A, 43 ± 2.5 vs. 43 ± 1.6, *p* = 0.78). Postoperatively, anxiety levels were reduced in both groups, with a significantly greater decrease observed in Group D on postoperative day 1 (Figure 3A, 41 ± 2.8 vs. 39 ± 2.1, *p* < 0.05). There was no difference in the EPDS scores between the two groups of patients on the sixth week after surgery (Figure 3B, 8.6 ± 1.6 vs. 7.9 ± 1.8, *p* = 0.29).

**Table 1:** Demographic data of the two groups.

Parameters	Group D	Group C
ASA grade	2 (2, 3)	2 (2, 3)
Age (year)	32.23 ± 5.11	31.47 ± 4.56
BMI (kg/m^2^)	27.00 ± 3.66	28.95 ± 5.20
Multipara *n* (%)	12 (35.3)	10 (29.4)
Gestational age (weeks)	38 (37, 39)	38 (38, 39)
Year of education *n* (%)		
6–9	3 (8.8)	3 (8.8)
9–12	2 (5.9)	2 (5.9)
12–16	24 (70.6)	24 (70.6)
>16	5 (14.7)	5 (14.7)
Hypotension (*n*, %)	3 (8.8)	3 (8.8)
Diabetes (*n*, %)	4 (11.4)	5 (14.7)

Values are expressed as median (interquartile range [IQR]), mean ± SD, and frequency (%).

ASA, American Society of Anesthesiologists; BMI, body mass index; Group D, the dexmedetomidine group; Group C, the control group.

**Table 2:** Intraoperative and postoperative data of the two groups.

Parameters	Group D	Group C	*p* value
Duration of surgery (min)	58.74 ± 16.16	59.62 ± 15.03	0.82
Duration of anesthesia (min)	80.38 ± 15.52	82.35 ± 17.70	0.63
24 h after surgery			
Pain scores at rest	1 (1, 2)	1 (1, 2)	0.67
Pain scores during activity	1 (1, 2)	2 (2, 3)	<0.001*
Consumption of PCEA (ml)	50 (48, 53.5)	56 (54, 60)	<0.001*
Length of postoperative hospital stay	4 (3, 4)	4 (3, 4)	0.86
Rescue analgesics case (Nalbuphine)	5	6	0.75
Antiemetics case (Azasetron or droperidol)	5	7	0.68
Fluid infusion volume (ml)	1852.3 ± 215.6	1826.7 ± 208.9	0.56
Intraoperative blood loss (ml)	125.5 ± 32.8	130.2 ± 35.1	0.48

Values are expressed as mean ± SD, median (interquartile range [IQR]), and frequency (%). *Significant difference (*P* < 0.001) between Group D and Group C.

PCEA, patient-controlled epidural analgesia; Group D, the dexmedetomidine group; Group C, the control group.

There was no difference in the pain scores at rest 24 h after surgery between the two groups of patients (Table 2 1 vs. 1, *p* = 0.67). The length of postoperative hospital stay of the two groups of patients was comparable (Table 2, 4 vs. 4, *p* = 0.86).

**Figure 4:**
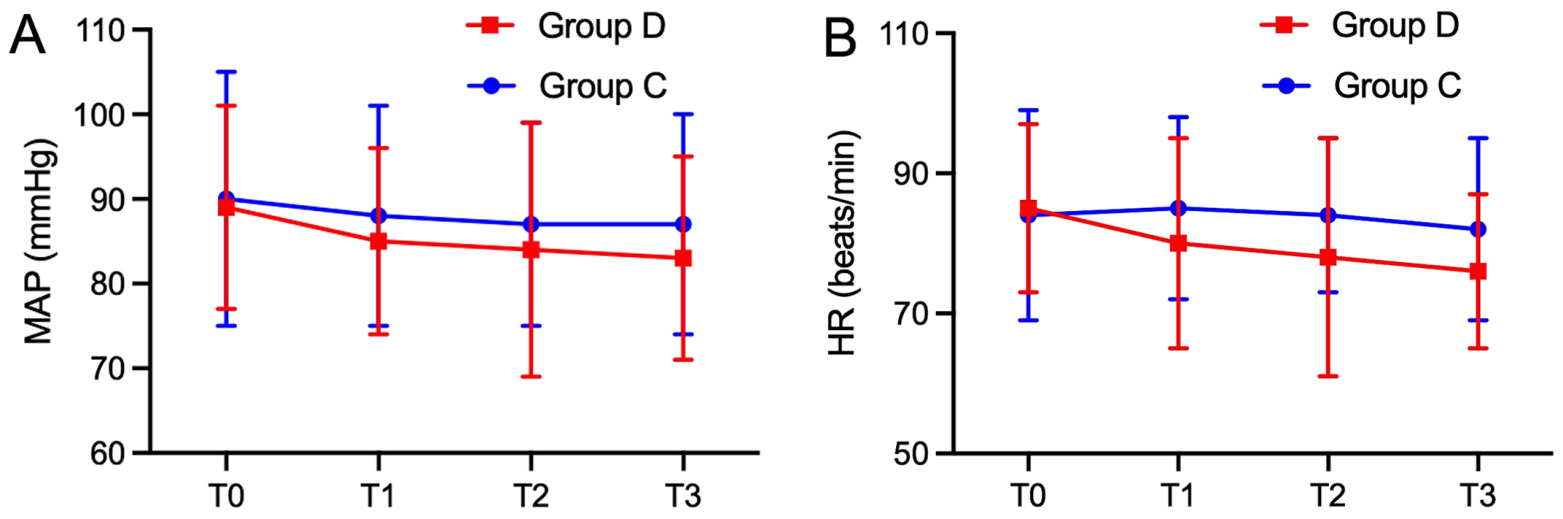
MAP **(A)** and HR **(B)** during dexmethetomidine or saline infusion. MAP and HR were not significantly different at each time point between the two groups. MAP, mean arterial pressure; HR, heart rate; T0, time before administration of dexmethetomidine or saline infusion; T1, 5 min after infusion; T2, 10 min after infusion; T3, 15 min after infusion.

To evaluate the maternal safety profile of the intervention, we analyzed and compared maternal MAP (Figure 4A, *p* = 0.17) and HR (Figure 4B, *p* = 0.11) during dexmedetomidine infusion. Although transient decreases in MAP and HR were noted in Group D during the infusion period, no statistically significant differences were observed relative to Group C, which validates the clinical safety of bolus dexmedetomidine administration in parturients undergoing cesarean section.

Six parturients in Group D and 6 in Group C received 10–15 mg nalbuphine to rescue insufficient neuraxial anesthesia (Table 2, *p* = 0.75). Prominent nausea and vomiting were observed in 7 parturients of the Group C and 5 of Group D (Table 2, *p* = 0.68); all affected cases were treated with azasetron or droperidol, leading to a significant resolution of symptoms. There was no significant difference in perioperative fluid infusion volume (Table 2, Group D: 1852.3 ± 215.6 mL; Group C: 1826.7 ± 208.9 mL, *p* = 0.56) and intraoperative blood loss (Table 2, Group D: 125.5 ± 32.8 mL; Group C: 130.2 ± 35.1 mL, *p* = 0.48) between the two groups.

Adverse events are summarized in Table 3. Shivering was observed in 6 patients (17.6%) in Group D and 18 patients (52.9%) in Group C (*p* < 0.001). Hypotension occurred in 3 patients (8.8%) in Group D and 2 patients (5.9%) in Group C (*p* = 0.75). No respiratory depression was reported in either group. Nausea and vomiting were noted in 7 patients (20.6%) in Group D compared with 5 patients (14.7%) in Group C (*p* = 0.67). Neonatal Apgar scores at 1-min (Table 3, Group D: 9.21 ± 0.58, Group C: 9.15 ± 0.62, *p* > 0.05) and 5-min (Table 3, Group D: 9.76 ± 0.32, Group C: 9.71 ± 0.35, *p* > 0.05), and NBNA scores were comparable between the two groups (Table 3, Group D: 39, Group C: 38 at 24 h after delivery, *p* = 0.173, Group D: 38.5, Group C: 38 at 48 h after delivery, *p* = 0.312), suggesting no adverse effects on neonatal safety.

**Table 3:** Adverse events and neonatal outcomes.

Parameters	Group D	Group C	*p* value
Incidence of intraoperative shivering (*n*, %)	6 (17.6)	18 (52.9)	<0.001*
Hypotension (*n*, %)	3 (8.8)	2 (5.9)	0.754
Respiratory depression (n, %)	0 (0)	0 (0)	—
Nausea/Vomiting (*n*, %)	2 (5.9)	4 (11.7)	0.67
Apgar score			
1-min	9.21 ± 0.58	9.15 ± 0.62	>0.05
5-min	9.76 ± 0.32	9.71 ± 0.35	>0.05
NABA, median (IQR)			
Day P 1	39 (3)	38 (2)	0.173
Day P 2	38.5 (3)	38 (2)	0.312

Values are expressed as frequency (%), mean ± SD and median (IQR). *Significant difference (*P* < 0.001) between Group D and Group C.

IQR, interquartile range; NABA, neonatal behavioral neurological assessment; Group D, the dexmedetomidine group; Group C, the control group.

## 4 Discussion

In the present study, we found that the intravenous bolus treatment of dexmedetomidine improved the sleep quality on the first night and the sixth week in parturients after cesarean section.

Sleep disturbance is a highly prevalent issue that plagues women throughout pregnancy and the postpartum period, with more than 60% of parturients reporting poor sleep quality in the early postpartum stage and over 40% suffering from compromised sleep quality accompanied by reduced total sleep duration (12, 14, 25). The sleep patterns and feeding methods of infants, the pain and complications of delivery, and changes in hormone secretion levels during pregnancy can all lead to shortened sleep duration and poor sleep quality in the early postpartum period (7, 16). New mothers undergo changes in their psychology, emotions, and social relationships. More critically, poor postpartum sleep is a well-recognized risk factor for postpartum depression, and this risk is exacerbated by the fact that 14–23% of new mothers already experience anxiety and depressive symptoms in the postpartum period, forming a vicious cycle of poor sleep and deteriorated mental health (4).

Notably, cesarean section patients experience more severe sleep disturbances, with sleep duration and quality peaking at their lowest in the immediate postpartum period and recovering gradually over six months (2, 26). This period of poorest sleep quality in new mothers further exacerbates their psychological distress, creating a detrimental cycle of poor sleep and impaired mental status (25). The present study was therefore designed to align with the natural trajectory of postpartum sleep changes in parturients, delivering targeted intervention during this most critical phase of sleep impairment (9). Even a mild improvement in sleep quality during this vulnerable period may promote postoperative recovery and support sustained long-term sleep improvement (11).

In our previous study, we demonstrated that intraoperative dexmedetomidine infusion enhanced sleep quality and elevated melatonin secretion in patients undergoing lobectomy during the first two postoperative nights (20). We hypothesize that dexmedetomidine exerts a favorable effect on postoperative melatonin production, which in turn contributes to improved sleep quality (20, 21). The present investigation extends this line of research to explore the potential efficacy of dexmedetomidine in women undergoing elective cesarean section, a population marked by high levels of anxiety and nervousness prior to fetal delivery. In our study, dexmedetomidine infusion was initiated immediately following fetal extraction, which served to calm maternal emotional tension, induce intraoperative sedation, and alleviate discomfort stemming from surgical stimulation. Additionally, dexmedetomidine is known to suppress the release of inflammatory mediators and inhibit sympathetic nervous system activity (27, 28), and these mechanisms, alongside its anxiolytic and sedative properties, may collectively account for the higher subjective sleep quality scores observed in Group D. Poor postpartum sleep quality is well-documented to correlate with elevated postpartum depression scale scores (7). While our findings confirm that dexmedetomidine yields an immediate improvement in postoperative sleep quality and anxiety, and that dexmedetomidine treatment significantly increased the sleep quality scores remained at sixth week postpartum, no significant between-group difference was noted in EPDS scores at this follow-up time point. These results suggest that the long-term benefits of dexmedetomidine monotherapy for maternal psychological outcomes may be limited, and that optimal long-term prognostic effects are likely to require adjunctive non-pharmacological interventions.

Neuraxial anesthesia is the standard anesthesia technique for parturients undergoing cesarean section. Shivering is the most common discomfort experience that new mothers complain about (29, 30). The incidence of shivering in parturients after neuraxial anesthesia is approximately 51.8%, usually occurring within 20 min after the anesthesia is completed (29, 31). The mechanism of its occurrence remains unclear. Potential factors include changes in the temperature regulation threshold, a decrease in core temperature, and the cooling effect of the fluid injected into the neuron axis, etc. (32, 33). Clinical interventions for neuraxial anesthesia-induced shivering include physical warming and pharmacological agents, and dexmedetomidine has been proven to have a significant anti-shivering effect in previous studies (30). This study further verified that the incidence of intraoperative shivering was significantly lower in the dexmedetomidine group (17.6%) than in the control group (52.9%) (*p* < 0.001), consistent with the results of previous research (31). The anti-shivering mechanism of dexmedetomidine may involve the regulation of the central thermoregulatory center in the hypothalamus: by activating *α*2-adrenergic receptors, it resets the thermoregulatory threshold, reduces the body’s response to hypothermia, and inhibits the shivering reflex (29, 30). The significant reduction in shivering incidence in Group D not only improved the intraoperative experience of parturients but also reduced the physiological stress caused by shivering, which is an important part of optimizing the perioperative management of cesarean section (20).

Compared with vaginal delivery, cesarean section is associated with more severe postoperative pain and negative emotions, which impair maternal breastfeeding motivation and recovery, and pain-induced sleep disturbance further forms a vicious circle (28, 32). As a highly selective α₂-adrenergic agonist, dexmedetomidine exerts pharmacodynamic synergism with ropivacaine-sufentanil PCEA in cesarean section parturients; it potentiates ropivacaine’s local anesthetic effect and sufentanil’s central analgesic action, reduces drug dosages, mitigates pain-induced sleep fragmentation, and independently modulates sleep–wake pathways to induce physiological non-rapid eye movement sleep without respiratory depression, complementing PCEA’s sleep-improving effects and reducing sleep-disrupting adverse events (e.g., nausea).

Clinical evidence corroborates the synergistic effects of dexmedetomidine in cesarean section patients. Wang et al. reported that perioperative dexmedetomidine administration promotes earlier initiation of exclusive breastfeeding, enhances postoperative recovery comfort, and optimizes analgesic efficacy in cesarean section parturients (22). Li et al. further verified that epidural dexmedetomidine supplementation improves subjective sleep quality, reduces PCEA utilization, and alleviates postoperative pain, which is closely related to its inherent sedative and analgesic properties (17). In the present study, intraoperative intravenous administration of dexmedetomidine significantly reduced activity-related pain scores in Group D compared with the Group C. The synergistic interaction between dexmedetomidine’s sedative-analgesic effects and PCEA intervention effectively improves postoperative sleep quality and optimizes recovery outcomes in parturients undergoing cesarean section.

Perioperative rescue analgesia and antiemetic interventions were administered in accordance with standard clinical protocols for inadequate neuraxial block and symptomatic nausea/vomiting, respectively. No statistically significant intergroup differences were observed in the utilization of such adjunctive medications, thus precluding their potential confounding effects on the primary study endpoint of maternal sleep quality. Given that surgical pain and nausea/vomiting are well-recognized critical factors contributing to postoperative sleep disturbance in cesarean section parturients, the rational administration of rescue analgesics and antiemetics is clinically instrumental in ameliorating maternal sleep quality by alleviating these sleep-disruptive conditions. The standardized and consistent application of adjunctive medication protocols across both study groups ensured uniformity in perioperative clinical management, which eliminated bias in the assessment of dexmedetomidine’s sleep-improving efficacy and enhanced the methodological rigor of the study. This uniformity confirms that the observed differences in postoperative sleep quality between the two groups were attributable exclusively to the intraoperative dexmedetomidine bolus intervention, rather than disparate use of adjunctive analgesics or antiemetics, thereby supporting the reliability of the study’s core conclusions regarding efficacy of dexmedetomidine.

The safety of pharmacological agents in obstetric practice is the primary consideration, especially regarding drug transfer into breast milk and potential adverse effects on newborns (18, 19). Although dexmedetomidine has been used for intraoperative sedation in parturients receiving neuraxial anesthesia, research on its transfer into human colostrum is limited, which is the main factor restricting its clinical application in postpartum women (18, 34). The present study confirmed the safety of low-dose intravenous dexmedetomidine (0.5 μg/kg) for both parturients and neonates from multiple aspects. First, for parturients: the incidence of adverse events such as hypotension (8.8% in Group D vs. 5.9% in Group C, *p* = 0.75) and nausea and vomiting (11.7% vs. 5.9%, *p* = 0.67) was not significantly different between the two groups, and no respiratory depression was recorded in either group. Bradycardia was effectively managed with atropine when it occurred, with no need for infusion cessation in any case. Second, for neonates, NBNA results were comparable between the two groups (*p* = 0.312), indicating no adverse effects of dexmedetomidine on neonatal neurological development and vital signs. Additionally, the dexmedetomidine dose used in this study (0.5 μg/kg) was lower than that in the study by Yoshimura et al., which reported that the milk-to-plasma ratio of dexmedetomidine in parturients was less than 1 and the relative infant dose was extremely low (18, 34). The single bolus infusion regimen further reduces the cumulative drug exposure of parturients, making breast milk transfer negligible. These results confirm that intraoperative intravenous bolus infusion of dexmedetomidine after fetal delivery is a safe regimen for both cesarean section parturients and their newborns.

Several limitations should be mentioned in our research. First, the optimal dose of dexmedetomidine for improving postpartum sleep quality remains to be detemined. This study only used a single low dose (0.5 μg/kg); future dose-ranging studies are needed to determine the minimum effective dose and the maximum safe dose that can improve long-term postpartum sleep outcomes without increasing adverse events. Second, the study did not measure the concentration of dexmedetomidine in maternal plasma and breast milk. Although previous research has confirmed a low milk-to-plasma ratio of dexmedetomidine, direct detection of drug concentrations in this study population would provide more direct and reliable evidence for the safety of the regimen, especially for breastfeeding nenates. Third, sleep quality was evaluated only by the subjective RCSQ, without objective sleep monitoring tools such as polysomnography (PSG). Subjective scores can reflect the self-perceived sleep quality of parturients, but objective monitoring can more accurately assess sleep architecture (e.g., sleep stages, sleep latency, and awakening times), which would make the research results more comprehensive and rigorous.

## 5 Conclusion

Intraoperative intravenous bolus dexmedetomidine administration improves postoperative sleep quality (1st night and 6-week follow-up) and alleviates 24-h post-surgery activity-related pain in parturients undergoing elective cesarean section. Given its administration post fetal delivery and favorable maternal-neonatal safety profile, this intervention serves as a safe and effective perioperative strategy to optimize obstetric anesthesia care and enhance maternal postoperative comfort in clinical practice.

## Data Availability

The original contributions presented in the study are included in the article/supplementary material, further inquiries can be directed to the corresponding authors.
